# From Prescription to Dependence? Retrospective Cohort Study of Long-term Benzodiazepine and Z-drug Use

**DOI:** 10.1192/j.eurpsy.2026.10423

**Published:** 2026-07-31

**Authors:** D. Kubek, M. Mišuth, M. Selvek, L. Kováčová, B. Saal, R. Mužik, D. Breznoščáková

**Affiliations:** 1 Dovera Health Insurance Company, Bratislava; 2 Department of Social and Behavioural Medicine, University of Pavol Jozef Safarik. Faculty of Medicine, Kosice; 3 Center for Psychical Functions, Vranov nad Topľou, Slovakia

## Abstract

**Introduction:**

Benzodiazepines and Z-drugs (BZDR) are commonly prescribed for anxiety and insomnia, yet their long-term use is discouraged due to risks of dependence and adverse outcomes. Evidence on long-term BZDR use in Slovakia remains scarce.

**Objectives:**

We aimed to describe BZDR prescription in Slovakia using administrative data from a health insurance company, focusing on long-term use, patient characteristics and differences between specialist and general practitioners (GP) prescription patterns.

**Methods:**

We conducted a retrospective cohort study using administrative data from a Slovak health insurance company covering approximately one-third of the Slovak population (1.7 million insurees in 2024). The study included all prescriptions with ATC codes N05BA, N05CD and N05CF (N = 5 144 826) between 2015 and 2024 for 343 280 patients insured at least 6 months before first prescription. Long-term use was defined as at least 3 consecutive prescriptions with gap of maximum 60 days in between and covering at least 90 daily doses. Statistical methods including linear regression and chi-square tests were applied.

**Results:**

The mean age at time of treatment initiation was 52.5 years, women represented 63.0% of the cohort. Criteria for long-term BZDR use were met by 82 553 (24.0%) patients at least once during the study period. Long-term users were older at time of treatment initiation (mean age 58.0 years), with slightly higher proportion of women (64.4%, p < 0.001). There was no significant linear trend in ratio of long-term users over the study period (p = 0.151).

GPs were responsible for initiating BZDR treatment in 63.9 % cases (219 349 patients). Among specialists, psychiatrists initiated treatment in most cases (51 985, 15.1%). The difference in ratio of long-term users across treatment initiating specialties was significantly different (p < 0.001): GPs (25.1%), psychiatrists (34.9%) and other specialties (13.0%).

**Conclusions:**

Long-term BZDR use is a prevalent phenomenon, affecting nearly one in four patients. Initiation of treatment by psychiatrists lead to long-term use more often compared to GPs and other specialties. Older patients and women showed increased susceptibility to long-term use of BZDR. These findings highlight the importance of monitoring treatment duration and developing strategies to prevent unnecessary long-term exposure to BZDR.

**Disclosure of Interest:**

None Declared

